# Heidihuangwan alleviates renal fibrosis in rats with 5/6 nephrectomy by inhibiting autophagy

**DOI:** 10.3389/fphar.2022.977284

**Published:** 2022-09-09

**Authors:** Ying-Ying Li, Zeng-Hui Tian, Guang-Hui Pan, Ping Zhao, De-Jun Pan, Jun-Qing Zhang, Li-Ying Ye, Fa-Rong Zhang, Xiang-Dong Xu

**Affiliations:** ^1^ College of First Clinical Medical, Shandong University of Traditional Chinese Medicine, Jinan, China; ^2^ Department of Nephrology, Affiliated Hospital of Shandong University of Traditional Chinese Medicine, Jinan, China; ^3^ Department of Nephrology, Tai’an City Hospital of Traditional Chinese Medicine, Tai’an, China; ^4^ Department of Clinical Laboratory, Affiliated Hospital of Shandong University of Traditional Chinese Medicine, Jinan, China; ^5^ College of Traditional Chinese Medicine, Shandong University of Traditional Chinese Medicine, Jinan, China; ^6^ Experimental Center, Affiliated Hospital of Shandong University of Traditional Chinese Medicine, Jinan, China

**Keywords:** heidihuangwan, renal fibrosis, autophagy, insulin-like growth factor 1, PI3K/Akt/mTOR

## Abstract

Renal fibrosis is a common pathway for the progression of various chronic kidney diseases (CKD), and the formation and deterioration will eventually lead to end-stage renal failure, which brings a heavy medical burden to the world. HeidihuangWan (HDHW) is a herbal formulation with stable and reliable clinical efficacy in the treatment of renal fibrosis. However, the mechanism of HDHW in treating renal fibrosis is not clear. In this study, we aimed to investigate the mechanism of HDHW to improve renal fibrosis. Wistar rats were randomly divided into the normal control group, 5/6 Nephrectomy group, astragaloside IV (AS-IV) group, HDHW group, and HDHW + IGF-1R inhibitor (JB1) group. Except for the normal control group, the rat renal fibrosis model was established by 5/6 nephrectomy and intervened with drugs for 8 weeks. Blood samples were collected to evaluate renal function. Hematoxylin-Eosin (HE), Periodic Acid-Schiff (PAS), Modified Masson’s Trichrome (Masson) staining were used to evaluate the pathological renal injury, and immunohistochemistry and Western blotting were used to detect the protein expression of renal tissue. The results showed that HDHW was effective in improving renal function and reducing renal pathological damage. HDHW down-regulated the levels of fibrosis marker proteins, including α-smooth muscle actin (α-SMA), vimentin, and transforming growth factors–β(TGF-β), which in turn reduced renal fibrosis. Further studies showed that HDHW down-regulated the expression of autophagy-related proteins Beclin1 and LC3II, indicating that HDHW inhibited autophagy. In addition, we examined the activity of the class I phosphatidylinositol-3 kinase (PI3K)/serine-threonine kinase (Akt)/mTOR pathway, an important signaling pathway regulating autophagy, and the level of insulin-like growth factor 1 (IGF-1), an upstream activator of PI3K/Akt/mTOR. HDHW upregulated the expression of IGF-1 and activated the PI3K/Akt/mTOR pathway, which may be a vital pathway for its inhibition of autophagy. Application of insulin-like growth factor 1 receptor (IGF-1R) inhibitor further confirmed that the regulation of autophagy and renal fibrosis by HDHW was associated with IGF-1-mediated activation of the PI3K/Akt/mTOR pathway. In conclusion, our study showed that HDHW inhibited autophagy by upregulating IGF-1 expression, promoting the binding of IGF-1 to IGF-1R, and activating the PI3K/Akt/mTOR signaling pathway, thereby reducing renal fibrosis and protecting renal function. This study provides support for the application and further study of HDHW.

## Introduction

Chronic kidney disease (CKD) is mainly characterized by irreversible impairment or loss of renal function, eventually leading to end-stage renal disease (ESRD) or chronic renal failure (CRF). It has become one of the serious global public health problems because of its high prevalence (GBD 2017 Disease and Injury Incidence and Prevalence Collaborators, 2018), high mortality (GBD 2017 Causes of Death Collaborators, 2018; GBD 2017 DALYs and HALE Collaborators, 2018), many complications (GBD Chronic Kidney Disease Collaboration, 2020), and lack of effective means of prevention and treatment (Zhang et al., 2022a). Renal fibrosis is the underlying pathological process of CKD independent of the underlying cause, and its formation and progression can lead to the destruction of normal kidney structure and loss of kidney function, eventually leading to end-stage renal failure (Liu et al., 2017). End-stage renal failure is difficult to reverse, and since most chronic kidney diseases lead to renal fibrosis, which in turn is an important pathological process leading to the continued progression of kidney damage. Therefore, there is a strong interest in identifying the underlying factors and pathogenesis of this process to reverse the progression of CKD (da Silva et al., 2015), and the development of new strategies for the treatment of this pathological process is considered to be extremely urgent for the prevention and treatment of CKD.

The pathogenesis of renal fibrosis is complex and has been outlined by some investigators as 4 pathological stages, mainly including Stage 1: Activation and injury stage. The renal disease leads to epithelial cell injury (E-calcine mucin shedding), fibroblast proliferation, and macrophage infiltration. Stage 2: Fibrogenic signaling stage. Inflammatory and fibrotic signaling pathways are activated. Stage 3: Fibrosis stage. Increased epithelial-mesenchymal transition (EMT) and extracellular matrix (ECM) deposition (EMT may also lead to ECM deposition). Stage 4: Destructive stage. Fibronectin, collagen, and α-SMA increase, eventually leading to renal failure (Zhuang et al., 2019). Multiple cellular targets and molecular pathways are involved in these stages, and the cellular targets mainly include the intrinsic renal cells such as podocytes (Xuan et al., 2021), tubular epithelial cells (Hu et al., 2022a), and thylakoid cells (Xiao et al., 2022), especially tubular epithelial cells, as interstitial myofibroblasts are the main effector cells in renal fibrosis, a large proportion of which are formed through the occurrence of EMT in tubular epithelial cells in the affected kidney (Hu et al., 2021). Molecular pathways mainly include nuclear factor-kappaB (NF-κB) (Wang et al., 2018a), TGF-β1/Smad (Loboda et al., 2016), Notch, wingless-type MMTV integration site (Wnt), Hedgehog (Edeling et al., 2016), protein kinase C (PKC)/extracellular regulated protein kinases (ERK), and PI3K/Akt (Liu et al., 2017), etc. Notably, related studies have shown that the PI3K/Akt signaling pathway can be used as an intervention target in renal fibrosis (Fan et al., 2022; Li et al., 2022), and also, the PI3K/Akt signaling pathway plays an important regulatory role in the process of cellular autophagy.

Autophagy is closely related to renal fibrosis, and a recent study found that sustained activation of autophagy is detrimental to the kidney after severe injury, leading to renal cell senescence and promoting renal fibrosis through the secretion of pro-fibrotic cytokines (Zheng et al., 2019). mTOR is one of the key regulators of autophagy in mammalian cells and can be activated by signal transducers including PI3K and Akt activation in response to insulin, IGF and other growth signals, which in turn inhibit autophagy (Liang et al., 2022). And further studies have shown that IGF-1, as a PI3K/Akt activator, improves renal fibrosis indicators in UUO rats (Wu et al., 2016). Therefore, targeting the IGF-1/PI3K/Akt/mTOR pathway to inhibit the sustained activation of autophagy during renal fibrosis may be an appropriate approach for the treatment of renal fibrosis.

HDHW, which consisted of Shu Di Huang, Cang Zhu, Gan Jiang, and Da Zao, is a classical kidney tonic herbal compound, and a large number of previous studies by our group have shown that HDHW can reduce renal fibrosis and improve renal function (Changlei et al., 2013) by improving micro inflammatory status, lowering lipids, improving renal anemia, and regulating gastrointestinal hormone disorders (Sun et al., 2014a; Sun et al., 2014b; Yang et al., 2016; Zhao et al., 2017; Yang et al., 2019), with significant clinical efficacy (Zhang et al., 2009; Changlei et al., 2013). However, the mechanism of HDHW for the treatment of renal fibrosis is not well defined due to the complex composition and targets of the Chinese medicine compound, and it deserves further study. In the present study, we identified the main components of HDHW using high-performance liquid chromatography and further evaluated the effects of HDHW on renal function and renal fibrosis in 5/6 nephrectomized rats. We hypothesized that HDHW could modulate the activity of the IGF-1/PI3K/Akt/mTOR pathway, inhibits autophagy and ameliorate renal fibrosis.

## Materials and methods

### Drugs and reagents

The single herbal granules (batch number 19051981) used in this study to prepare the decoction of HDHW were purchased from the Affiliated Hospital of Shandong University of Traditional Chinese Medicine (Manufactured by Jiangyin Tianjiang Pharmaceutical Co., Ltd., Wuxi, Jiangsu, China). The formula granules of HDHW have been qualified by Jiangyin Tianjiang Pharmaceutical Co., Ltd., whose quality inspection center has been accredited by CNAS National Laboratory. The Chinese herbal formula granules are single herbal products that simulate the decoction of traditional Chinese medicine tonics and are refined from Chinese herbal tablets by extraction, concentration, and drying processes using modern science and technology. AS-IV and Carboxymethyl Cellulose (CMC)were purchased from Chuzhou Sinoda Biotechnology Co. IGF-1R inhibitor (JB1) (78617-10-4) was purchased from Topscience Co. Ltd., Shanghai, China, with a purity of ≥98% (HPLC). Antibodies against PI3K (4249), Akt (4691), p-Akt (4060) were purchased from Cell Signaling Technology (Danvers, MA, United States). Antibodies against p-mTOR (80596-1-RR), LC3I/II (14600-1-AP), α-SMA (14395-1-AP), IGF-1 (28530-1-AP) were from Proteintech (Wuhan, China). Antibodies against p-PI3K(AP0854), and Beclin1 (A17028) were from ABclonal (Shanghai, China). Antibodies against mTOR (ab134903) were from Abcam (Cambridge, United Kingdom). Horseradish enzyme-labeled goat anti-rabbit IgG (H+L) was purchased from Beijing Zhongsun Jinqiao Biotechnology Co.

### Preparation of HeidihuangWan decoction

HDHW consists of Shu Di Huang, Cang Zhu, Gan Jiang, and Da Zao, and the herb information and composition ratio are shown in Table 1. The adult dosage of HDHW is 18.5g/60 kg. According to the equivalent dose ratio (6.3) converted from the body surface area of humans and rats, the dosage for rats is 1.94 g/kg. According to this ratio, the single herb granules were mixed and dissolved in ultrapure water. The decoction of HDHW was prepared. Some of the HDHW decoction was stored at −80°C and analyzed by UHPLC-Q-Orbitrap-MS/MS.

**TABLE 1:** **|** Composition of HeidihuangWan.

Chinses name	Scientific name	Plant part	Batch number	Adult daily dose of granules (g)	Corresponding	Composition (%)
		Used			Herb dose (g)	
Shu Di Huang	Rehmannia glutinosa (Gaetn.) Libosch. ex Fisch. et MeyPraeparata	Root	19051981	8	20	43.24
Cang Zhu	Atractylodes lancea (Thunb.)DC.	Rhizome	19070691	6	20	32.43
Gan Jiang	Zingiber oj-jicinale Rosc	Rhizome	19052411	0.5	3	16.22
Da Zao	Ziziphus jujuba Mill	Fruit	19060131	4	20	21.62

### UHPLC-Q-orbitrap-MS/MS analysis

Add 1,000 µL of 80% methanol to 200 µL of the solution of HeidihuangWan Decoction, and vortex for 10 min. Centrifuge at 12,000 rpm for 10 min at 4°C. The supernatant was filtered and analyzed on the machine. Mass spectrometry conditions: ion source: electrospray ionization source (ESI); Scan mode: positive and negative ion switching scan; Detection mode: full mass/dd-MS2; Resolution: 70000 (full mass); 17500 (dd-MS2); Scan range: 100.0–1500.0 m/z; Electrospray voltage (Spary Voltage: 3.2 kV (Positive); Capillary Temperature: 300°C; Collision gas: high-purity argon (purity ≥99.999%); Collision energy (N)CE: 30; Sheath gas: nitrogen (purity ≥99.999%), 40 Arb; Auxiliary gas: nitrogen (purity ≥99.999%), 15 Arb, 350°C; Data acquisition time: 30.0 min. Chromatographic column: AQ-C18, 150 × 2.1 mm, 1.8 µm, Welch; Flow rate: 0.3 ml/min; Aqueous phase: 0.1% formic acid/water solution; Organic phase: methanol; Eluent: methanol; Column oven temperature: 35°C; Autosampler temperature: 10.0°C; Autosampler injection volume: 5.00 µL see Table 2 for the gradient elution procedure. The data for the high-resolution liquid mass acquisition was completed by CD2.1 (Thermo Fisher). Retention time correction, peak identification, peak extraction, peak integration, and peak alignment were performed. The data were then searched and compared through the mzCloud database.

**TABLE 2:** Chromatographic gradient.

Time (min)	Water phase ratio (%)	Organic phase ratio (%)
1	98	2
5	80	20
10	50	50
15	20	80
20	5	95
27	5	95
28	98	2
30	98	2

### Animal experiments

#### Animals

Seven-week-old male Wistar rats (200 ± 20 g) were used as research subjects. All animals were purchased from Beijing Viton Lihua Laboratory Animal Science and Technology Co. Ltd. (SCXK 2016-0006). All animal experiments were conducted in compliance with the National Institute of Health Guide for the Care and Use of Laboratory Animals and were approved by the Animal Ethics Committee of Shandong University of Traditional Chinese Medicine (SDUTCM20201019002).

#### Making animal models

According to the method of the literature (Ji and HE, 2019), after adaptive feeding of the rats for 1 week, the rats were anesthetized by intraperitoneal injection of sodium pentobarbital (35 mg/kg). Then the rats were fixed in a prone position, the skin was sterilized routinely, the right kidney was exposed, the perirenal membrane was stripped, the renal phylum was ligated, the right kidney was removed (the adrenal gland was preserved), and finally, the skin was sutured layer by layer. Ten days after the first operation, after anesthetizing the rat in the same manner, the renal parenchyma of the upper and lower poles of the left kidney was removed, followed by hemostasis using gelatin sponge compression. Finally, the abdominal cavity was closed. In addition, the normal control group did not remove the kidney, and only the perirenal membrane was stripped. Penicillin was injected intraperitoneally for 3 consecutive days after surgery to prevent infection (Figure 2A).

#### Grouping and drug administration

After 12 weeks of modeling, blood was collected through the tail vein of rats to detect serum creatinine (Scr) and blood urea nitrogen (BUN) levels, and the success of modeling was judged by the fact that the Scr and BUN in the operated rats were significantly higher than those in the normal group. The rats with successful modeling were randomly divided into the following groups: 5/6 Nephrectomy group, HDHW group, AS-IV group, HDHW + IGF-1R inhibitor (JB1) group, and another normal control group (*n* = 6). HDHW group was given HDHW decoction, AS-IV group was given AS-IV decoction (1.5 mg/kg) dissolved in 0.5% CMC (Zhu et al., 2014). The corresponding volume of saline was given to the 5/6 Nephrectomy group and normal control group. The HDHW + JB1 group was injected subcutaneously with JB1 at a concentration of 50 ng/uL for 7 consecutive days, with each injection dose of 18 ng/g, and given HDHW decoction. The oral administration time of each group was 10:00a.m. every day, and the injection time of JB1 was 9:00a.m. every day, while the corresponding volume of saline was injected at the same time in the remaining groups to ensure the accuracy of the experiment. After 8 weeks of continuous administration, the rats were anesthetized by intraperitoneal injection of sodium pentobarbital (35 mg/kg), blood was collected from the abdominal aorta, and the kidneys were immediately isolated and part of them was stored in the refrigerator at −80°C, and the other part was immersed in 4% paraformaldehyde for fixation.

### Detection of serum biochemical indicators

After the end of the drug intervention, blood was collected through the abdominal aorta and the collected blood was left at room temperature for 2h, centrifuged at 3000 rpm for 20min, collected supernatant, and detected Scr, BUN, aspartate aminotransferase (AST) and alanine aminotransferase (ALT) in rat serum according to the manufacturer’s instructions.

### Histological and immunohistochemical assays

The kidney tissue soaked in 4% paraformaldehyde-fixed for 48 h was cut into small pieces, dehydrated, embedded with paraffin wax, and cut into 4 μm slices. Perform hematoxylin-eosin (HE), Masson, periodic-acid Schiff (PAS), and immunohistochemistry (IHC) staining experiments according to the manufacturer’s instructions. All sections were analyzed under an x200 advanced upright fluorescence microscope (Axio Imager. A2, ZEISS, Germany).

#### Hematoxylin-eosin staining

Dewax and hydrate to water. Stain nucleus with Hematoxylin Solution for 10 min.Rinse in running tap water. Differentiate with Differentiation Solution for 3 min, wash with tap water twice for 2 min each. Re-dyeing with Eosin Y Aqueous Solution for 1min. Quickly wash in deionized water. Dehydrated and transparent, then sealed with mounting medium. The nucleus is purple-blue, and the cytoplasm, basal membrane, and interstitial collagen are red to varying degrees.

#### Modified masson’s trichrome staining

Dewax and hydrate to water. Incubate the section into Mordant Solution in a 60°C incubator for 1 h, and wash with running water. Drop Celestite Blue Solution onto the section and stain for 2 min. Slightly wash with distilled water twice, each for 20 s. Drop Mayer Hematoxylin Solution onto the section and stain for 2 min. Slightly wash with distilled water twice, each for 20 s. Differentiate by Acid Alcohol Differentiation Solution for several seconds. Rinse in running water for 10min. Drop Ponceau-Acid Fuchsin Solution onto the section and stain for 10min. Slightly wash with distilled water twice, each for 20 s. Treat with Phosphmolybic Acid Solution for 10 min. Discard the remaining dye solution and directly stain with Aniline Blue Solution for 5 min without washing with water. As the radio of 1:2, mix the Acetic Acid Solution and water to prepare the Acetic Acid working Solution, and rinse in the Acetic Acid working Solution for 2 min. Dehydrate quickly in 95% ethanol. Dehydrated and transparent, sealed with mounting medium. The nucleus is dark red, the collagen fibers are blue, and the muscle fibers, cytoplasm, and keratin are red.

#### Periodic acid-schiff staining

Dewax and hydrate to water. Rinse in tap water for 2 min, and wash with distilled water twice. Place in Oxidant for 6 min at room temperature. Wash with tap water once, and then wash with distilled water twice. Soak the section in Schiff Reagent in a dark place at room temperature, and stain for 15 min. Rinse in tap water for 10 min. Stain with Mayer Hematoxylin Solution for 1 min. Differentiate by Acidic Differentiation Solution for 3 s. Wash with tap water for 12 min to blue. Dehydrated and transparent, sealed with mounting medium. The nuclei are blue, and the basal membrane, collagen fibers, muscle fibers, and cytoplasm are red.

#### Immunohistochemical staining

Dewax and hydrate to water. Sections were submerged in citric acid antigen repair solution and heated in a microwave oven for antigen repair. Wash 3 times with PBS for 5 min each time. Sections were incubated in 3% H_2_O_2_ solution for 25 min at room temperature and protected from light. Wash 3 times with PBS for 5 min each time. The serum was closed for 30 min. Thereafter, the sections were incubated with anti-Beclin1 (1:100), anti-LC3 (1:500), anti-α-SMA (1:2000), anti-TGF-β (1:500), and anti-Vimentin (1:300), and incubated overnight at 4°C. The sections were washed 3 times with PBS for 5 min each time. Add secondary antibody (1:200) dropwise and incubate at room temperature for 50 min. Wash 3 times with PBS for 5 min each time. Add DAB working solution dropwise on the sections until a brownish-yellow positive signal is observed under the microscope. Rinse the sections with water to terminate the color development. The hematoxylin staining solution was used to re-stain the nuclei for 3 min, and the sections were rinsed with water. Fractionated with hematoxylin differentiation solution for 3s and rinsed with water. Hematoxylin blue-returning solution was used to return the blue color and rinsed with water. Conventional dehydration and transparency, sealed with mounting medium. Positive sites were brownish-yellow. Cumulative optical density was collected and calculated using Image-Pro Plus software (Media Cybernetics, United States).

### Western blot analysis

Weigh the frozen kidney tissue and add 500 µL of tissue lysate (containing 5 µLPMSF, 5 µL phosphatase inhibitor) per 50 mg of kidney tissue and homogenize in a tissue homogenizer. The homogenized samples were continued to be lysed in ice water for 20 min and centrifuged at 12,000 rpm for 15 min at 4°C. The supernatant was collected and set aside at 4°C. Protein concentration was measured using the BCA Protein Quantitative Assay Kit. Mix SDS-PAGE protein loading buffer with protein samples at a ratio of 1:4, and adjust the protein sample concentration to 6 μg/μL by adding PBS. Heat at 100°C for 5 min to denature the protein sufficiently. 13 ul of the sample was uploaded to the gel for electrophoresis. The proteins in the gel were then transferred to PVDF membranes. 5% skim milk was closed at room temperature for 2 h. The PVDF membranes were placed in primary antibody working solution (anti-PI3K 1:1000, anti-Akt 1:1000, anti-mTOR 1:10000, anti-p-Akt 1:2000, anti-p-PI3K 1:1000, anti-p-mTOR 1:10000, anti-LC3I/II 1:2000, anti-Beclin1 1:1500, anti-α-SMA 1:2000, anti-IGF-1 1:1000), incubated overnight at 4°C. Then, PVDF membranes were co-incubated with secondary antibodies (1:2500) at room temperature for 1 h. Finally, enhanced chemiluminescence reagents were added to the PVDF membranes and the target proteins were visualized by a chemiluminescence imaging system (GE Amersham Imager600, United States).

### Data analysis

To ensure the accuracy of the experimental results, all experiments in this study were independently repeated 3 times. All statistical analyses were performed using SPSS 26.0 statistical software (IBM, United States). Statistical analysis among multiple groups was performed by one-way analysis of variance (ANOVA) followed by least significant difference (LSD) test or Dunnett’s T3 test. Statistical analysis between the two groups was performed by independent samples *t*-test. All data were expressed as means ± standard deviations. When *p* < 0.05, the result was considered to be statistically significant. Graphs were constructed using GraphPad Prism 6.0.

## Results

### Composition analysis of HeidihuangWan decoction

**FIGURE 1:**
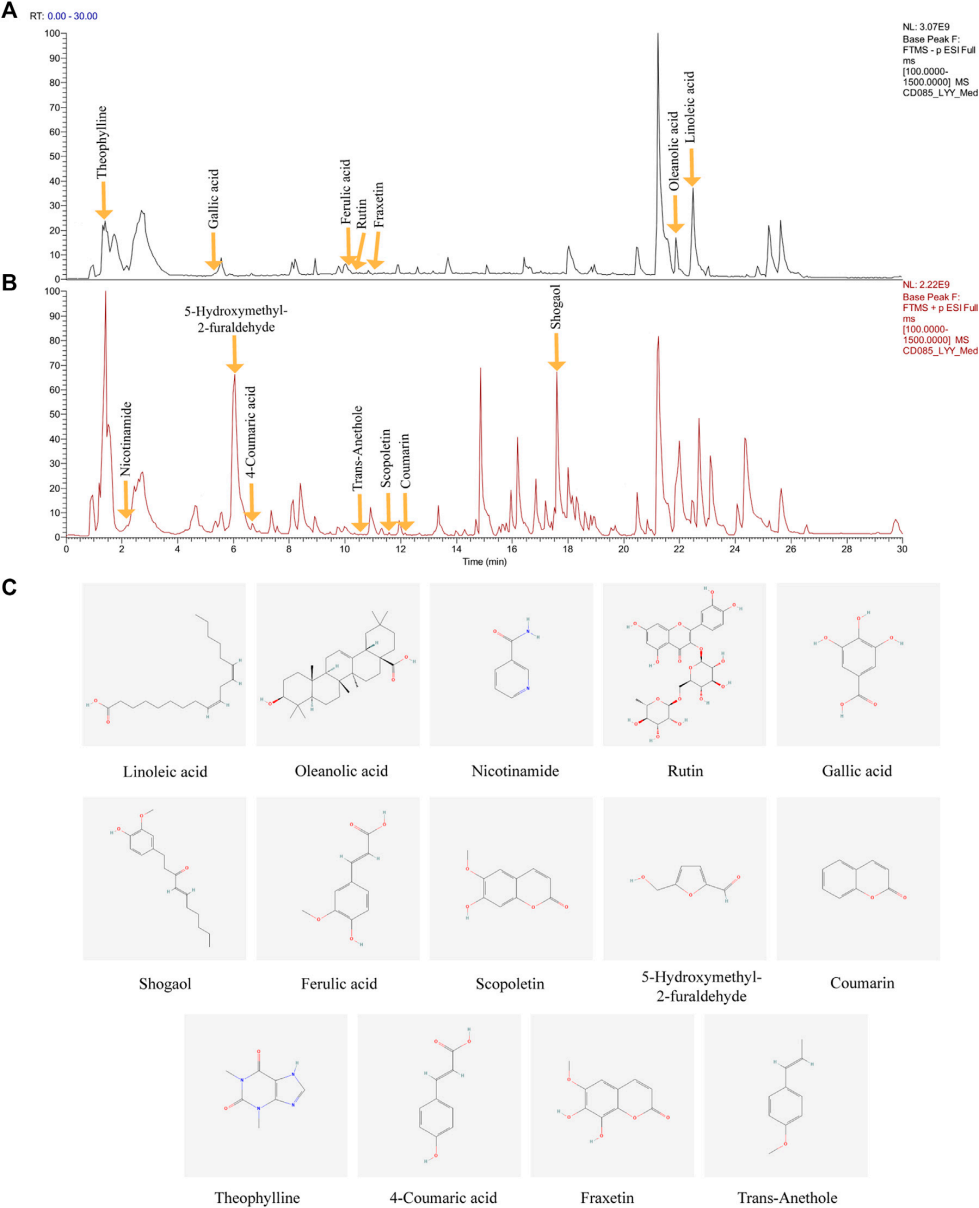
Identification of chemical components of HeidihuangWan decoction (HDHW). HDHW samples were examined by UHPLC-Q-Orbitrap-MS/MS. Total ion chromatography in negative **(A)** and positive **(B)** ion modes for HDHW samples are shown. **(C)** Molecular structure of constituents. UHPLC-Q-Orbitrap-MS/MS, Ultra High Performance Liquid Chromatography-Quadrupole-Electrostatic Field Orbitrap Tandem Mass Spectrometry.

The major compounds in the HDHW samples were identified by UHPLC-Q-Orbitrap-MS/MS analysis. The total negative **(** Figure 1A) and positive (Figure 1B) ion chromatograms of HDHW demonstrated the chemical composition of all compounds. Several components were found in HDHW. As shown in Figure 1C, fourteen compounds were distinguished: Linoleic acid, Oleanolic acid, Nicotinamide, Rutin, Gallic acid, Shogaol, Ferulic acid, Scopoletin, 5-Hydroxymethyl-2-furaldehyde, Coumarin, Theophylline, 4-Coumaric acid, Fraxetin, and Trans-Anethole. Then, we compared these components through the Traditional Chinese Medicine Systems Pharmacology Database and Analysis Platform (TCMSP) and found that Ferulic acid and 5-Hydroxymethyl-2-furaldehyde are the active components of Rehmannia glutinosa. Linoleic acid and Shogaol are the active ingredients of dried ginger. 4-Coumaric acid, Coumarin, Oleanolic acid, and Rutin are the active ingredients of jujube. We did not directly find the active ingredients of Atractylodes Rhizoma but found the ingredients often contained in aromatic medicines such as trans-Anethole, Oleanolic acid, and so on. In addition, the chemical structural formula, molecular weight, retention time, and peak area of these 14 components are shown in Table 3.

**TABLE 3:** **|** Information on the fourteen chemical constituents of HDHW.

Name	Formula	Calc.MW	m/z	RT (min)	Compound peak area	Corresponding Chinese medicine
Linoleic acid	C18 H32 O2	280.24037	279.23309	22.216	318419116.3	Rehmannia Ginger
Oleanolic acid	C30 H48 O3	456.36027	455.35327	22.212	35474426.59	Jujube
Nicotinamide	C6 H6 N2 O	122.04828	123.05556	2.162	975771717.5	--
Rutin	C27 H30 O16	610.15316	609.14606	12.92	284463906.2	Jujube
Gallic acid	C7 H6 O5	170.02058	169.01329	5.251	288677199.2	Jujube
Shogaol	C17 H24 O3	276.17209	277.17935	17.609	7393025189	Ginger
Ferulic acid	C10 H10 O4	134.03601	193.04982	11.873	173170124.2	Rehmannia
Scopoletin	C10 H8 O4	192.04215	193.04959	11.504	263234163.8	--
5-Hydroxymethyl-2-furaldehyde	C6 H6 O3	126.03187	127.03912	6.036	25644994018	Rehmannia
Coumarin	C9 H6 O2	146.03671	147.04399	12.159	371288074.8	Jujube
trans-Anethole	C10 H12 O	148.08878	149.09605	10.624	100703628.5	--
Theophylline	C7 H8 N4 O2	180.06244	179.05499	1.337	5872606281	--
4-Coumaric acid	C9 H8 O3	164.04747	165.05476	6.63	210576680.5	Jujube
Fraxetin	C10 H8 O5	208.03659	207.02931	11.563	133322185.3	--

### HeidihuangWan protects the renal function of 5/6 nephrectomy rats and reduces the pathological damage to the kidney

**FIGURE 2:**
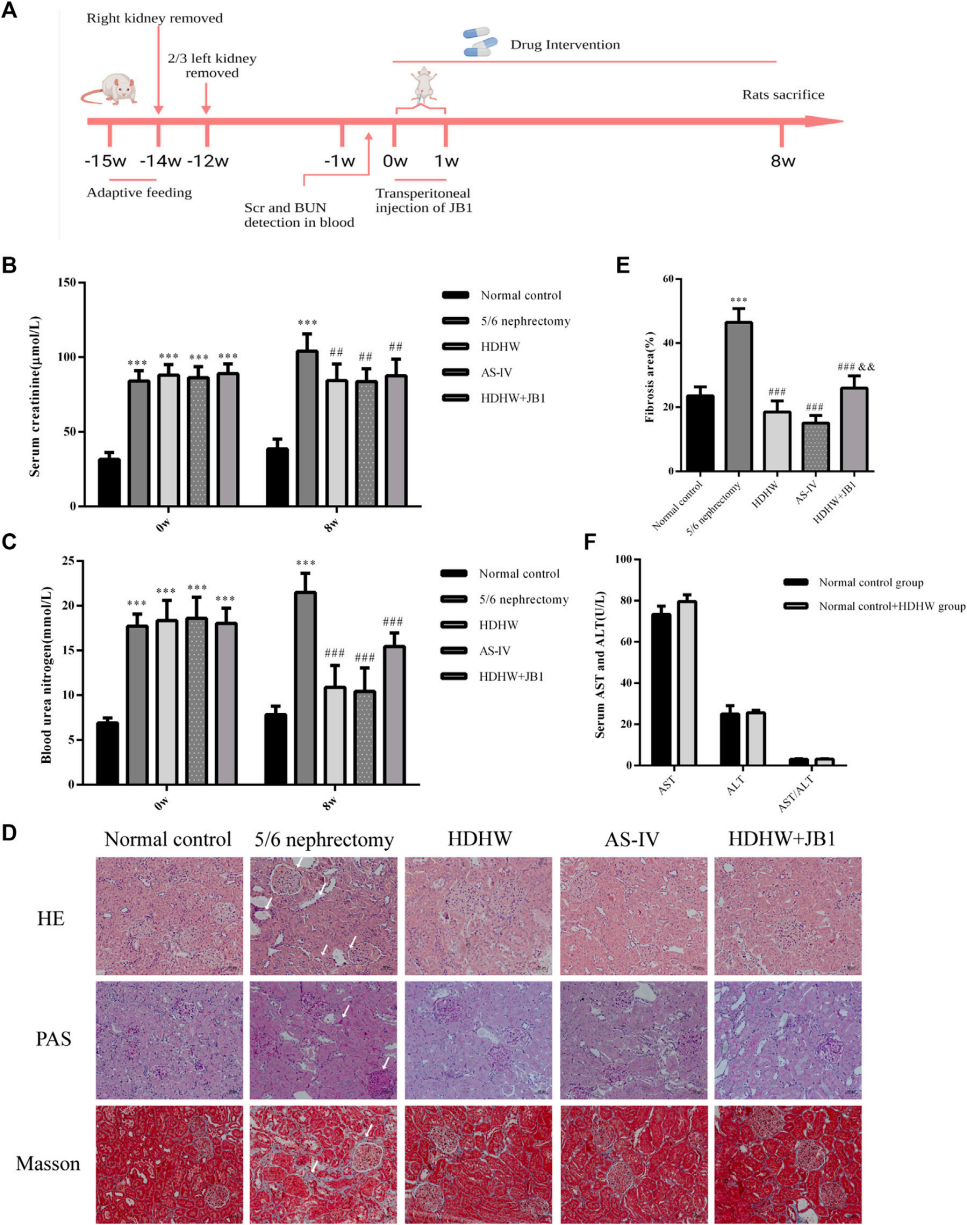
HeidihuangWan (HDHW) improves renal function and renal pathological damage in 5/6 nephrectomized rats. **(A)** Timeline of animal experiments. **(B)** Serum creatinine (Scr) and **(C)** blood urea nitrogen (BUN) levels before and after 8 weeks of drug intervention. **(D)** Hematoxylin-Eosin (HE), Periodic Acid-Schiff (PAS), Modified Masson’s Trichrome (Masson) staining. Collagen fiber deposition was observed in the 5/6 nephrectomy group (arrows). **(E)** The degree of renal fibrosis was calculated using ImageJ software. **(F)** Serum aspartate aminotransferase (AST) and alanine aminotransferase (ALT) levels after drug intervention. Data are presented as mean ± SD, *n* = 6. The data are expressed as ***p < 0.001: compared with normal control group; ##*p* < 0.01, ###*p* < 0.001: compared with 5/6 nephrectomy group; &&*p* < 0.01: compared with HDHW group, respectively.

ALT and AST levels were detected in the normal control group and normal control+HDHW group to evaluate the safety of HDHW. The results showed that there was no significant difference in AST, ALT, and AST/ALT between the normal control group and the normal control+HDHW group (*p* > 0.05) (Figure 2F), which proved that HDHW had no hepatotoxicity. The levels of Scr and BUN were detected before administration of the rats to verify whether the 5/6 nephrectomy rat model was successfully constructed. Then, the levels of Scr, BUN, and renal pathological changes were detected after drug administration to verify the effect of HDHW on renal function in 5/6 nephrectomy rats. The results showed that after 12 weeks of modeling, the levels of Scr and BUN in the 5/6 nephrectomy group, HDHW group, AS-IV group, and HDHW+JB1 group were significantly increased (*p* < 0.001) (Figures 2B,C), compared with the normal control group. It indicated that the 5/6 nephrectomy rat model was successfully constructed in this study; After 8 weeks of drug intervention, the levels of Scr and BUN in the 5/6 nephrectomy group were significantly increased compared with the normal control group (*p* < 0.001); Compared with 5/6 nephrectomy group, Scr and BUN levels in HDHW group, AS-IV group were significantly decreased (*p* < 0.01) (Figures 2B,C). The renal pathological staining results (Figure 2D) showed that the size and shape of the glomeruli in the normal control group were normal, with clear borders; The internal brush border structure of the renal tubules was intact. In the 5/6 nephrectomy group, the glomeruli were irregular in shape, with balloon adhesion, interstitial widening, proximal tubular atrophy, distal tubular dilatation with enlarged lumen, and vacuolation of tubular epithelial cells, accompanied by the infiltration of inflammatory cells in the renal interstitium and the proliferation of fibrous tissue was obvious. Compared with the 5/6 nephrectomy group, HDHW and AS-IV could alleviate these pathological lesions.

### HeidihuangWan alleviates renal fibrosis

**FIGURE 3:**
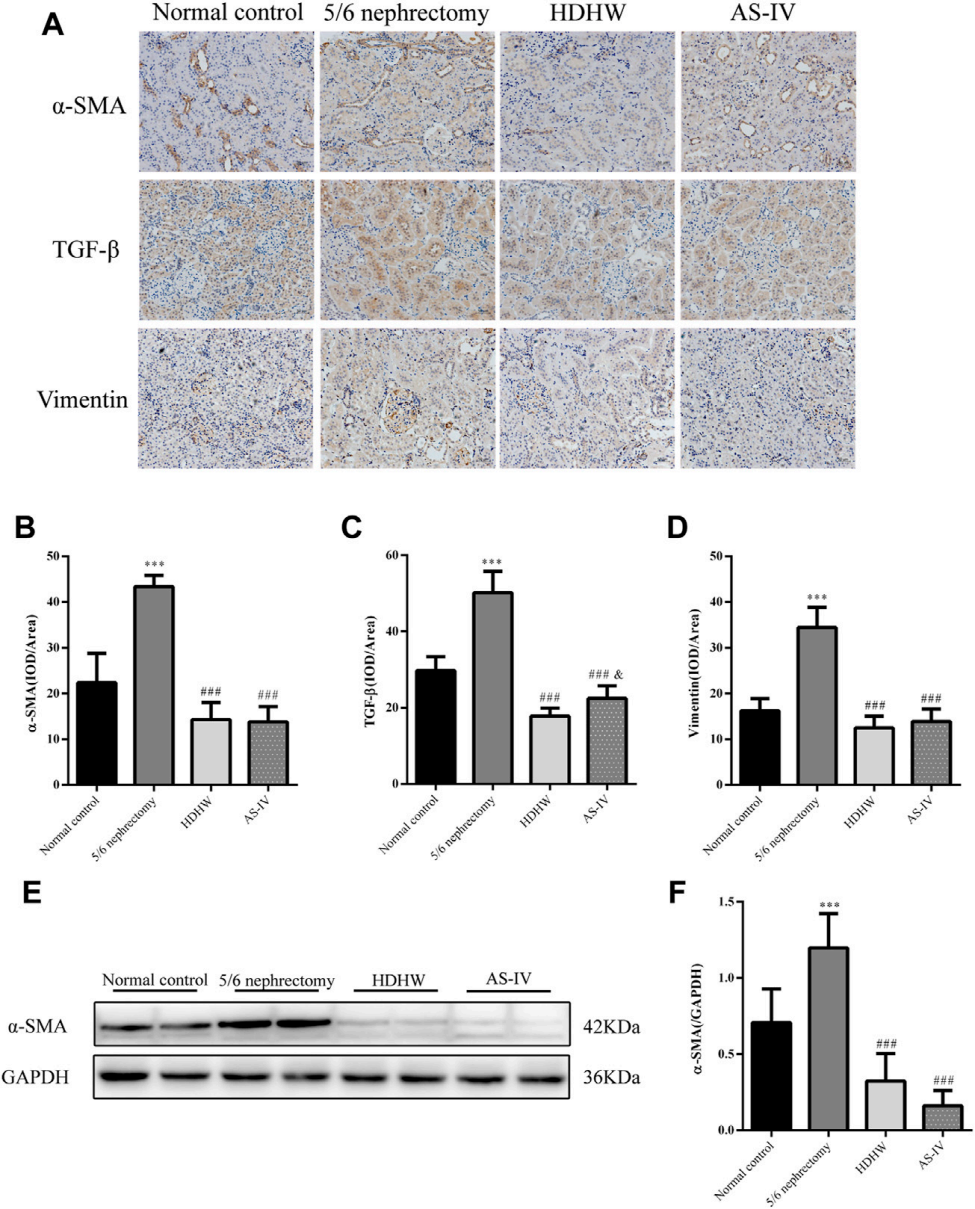
HeidihuangWan (HDHW) ameliorates renal fibrosis. **(A)** Immunohistochemical staining was used to analyze the expression of α-smooth muscle actin (α-SMA), transforming growth factor-β (TGF-β), and Vimentin. **(B,C,D)** Image-Pro Plus software was used to statistically analyze the immunohistochemical staining results of α-SMA, TGF-β, and Vimentin, respectively. **(E)** Western Blotting to detect the protein level of α-SMA. **(F)** Protein concentration analysis. Data are presented as mean ± SD, *n* = 6. The data are expressed as ***p < 0.001: compared with normal control group; ###*p* < 0.001: compared with 5/6 nephrectomy group; &*p* < 0.05: compared with HDHW group, respectively.

The pathological manifestations of renal fibrosis are dominated by increased collagenous components in the renal interstitium, tubular atrophy and dilated deformation, myofibroblast proliferation, and excessive accumulation of extracellular matrix. α-SMA, TGF-β, and Vimentin are fibrosis marker proteins whose expression is positively correlated with the degree of renal fibrosis (Ma et al., 2019). Among them, α-SMA, a marker of fibroblasts, also showed a positive correlation with extracellular matrix expression (Wan et al., 2020), which is a reliable test to assess the degree of renal fibrosis. To assess the effect of HDHW on renal fibrosis, we performed Masson staining and immunohistochemical staining for α-SMA, TGF-β, and Vimentin of rat kidney tissues, and further detected the level of α-SMA by Western blot. In Masson staining, red color indicates muscle fibers and blue color indicates collagen fibers. Compared with the normal control group, collagen fibers accumulated significantly in the renal tissue of the 5/6 nephrectomy group (*p* < 0.001); HDHW and AS-IV significantly reduced the number of collagen fibers compared with the 5/6 nephrectomy group (*p* < 0.001), which in turn improved renal fibrosis **(** Figures 2D,E). In addition, the expressions of α-SMA, TGF-β, and Vimentin in the kidney tissues of rats in the 5/6 nephrectomy group were significantly higher compared with the normal control group (*p* < 0.001); HDHW and AS-IV significantly down-regulated the expressions of α-SMA, TGF-β, and Vimentin compared with the 5/6 nephrectomy group (*p* < 0.001), and HDHW was more advantageous in downregulating TGF-β compared with the AS-IV group (*p* < 0.05) **(** Figure 3).

### HeidihuangWan inhibits autophagy

**FIGURE 4:**
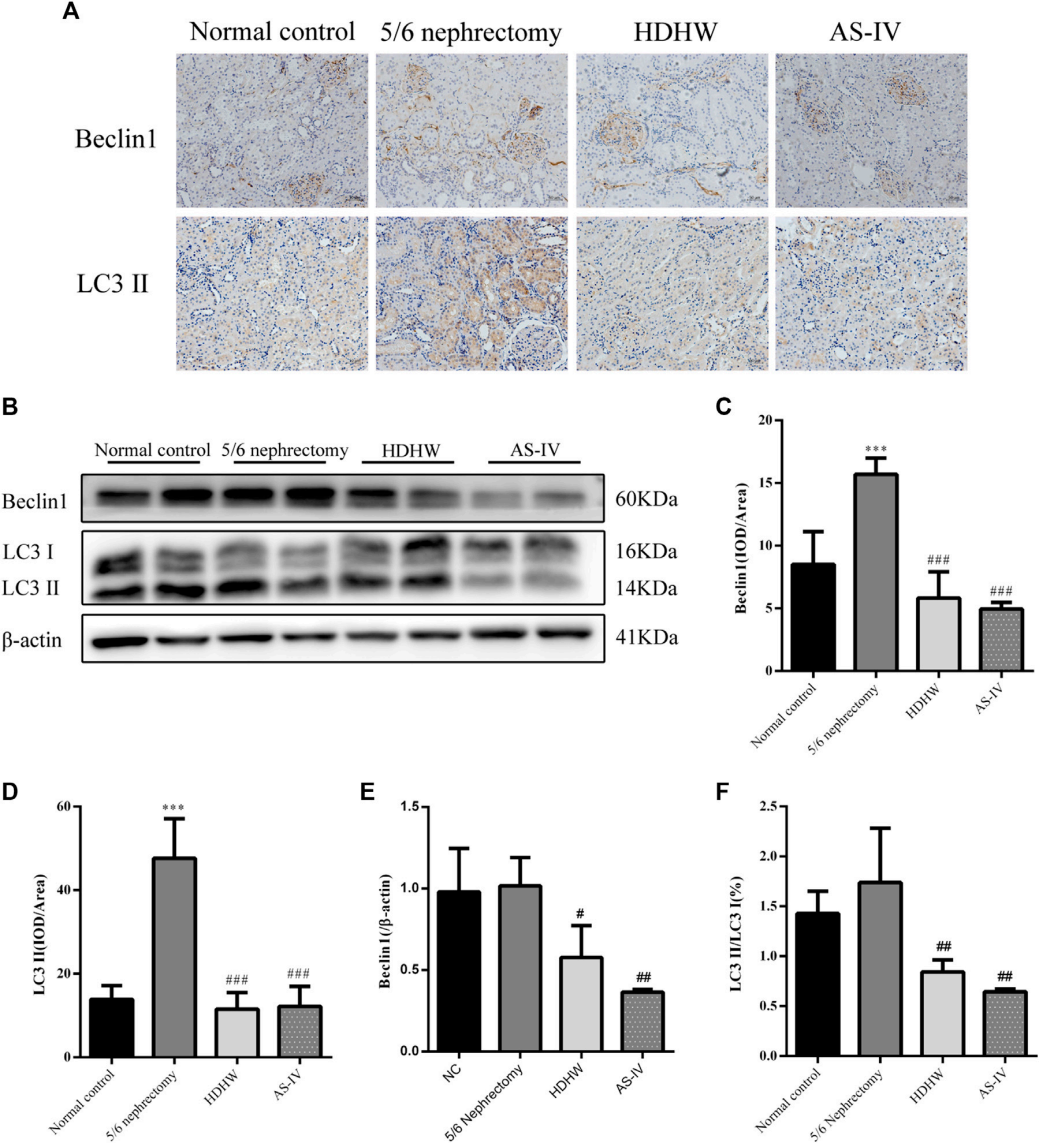
Effects of HeidihuangWan (HDHW) on autophagy. **(A)** Immunohistochemical staining analysis of Beclin1, LC3II expression. **(B)** Western Blotting to detect the protein levels of Beclin1, LC3II. **(C,D)** Image-Pro Plus software was used to statistically analyze the immunohistochemical staining results of Beclin1 and LC3II, respectively. **(E,F)** Protein concentration analysis. Data are presented as mean ± SD, *n* = 6. The data are expressed as ***p < 0.001: compared with normal control group; #*p* < 0.05, ##*p* < 0.01, ###*p* < 0.001: compared with 5/6 nephrectomy group, respectively.

Beclin1 (BECN1, Vps30 or Atg6) is a key component of the autophagy-stimulating complex. Beclin1 is involved in the initiation of autophagosome formation by forming a multiprotein complex. Microtubule-associated protein 1 light chain 3 (LC3) is cleaved to LC3I immediately after synthesis. Cytoplasmic LC3I is then recruited to the membrane and converted to LC3II after coupling to phosphatidylethanolamine during autophagosome formation. Increased conversion of LC3I to LC3II provides evidence for autophagy in mammalian cells (Li et al., 2010). To assess the effect of HDHW on autophagy, we examined the levels of autophagy marker proteins Beclin1, LC3II/Ⅰ in rat kidney tissue by Western blot and immunohistochemistry. Immunohistochemical analysis showed that the expression of Beclin1 and LC3II/Ⅰ was significantly upregulated in the 5/6 nephrectomy group, compared with the normal control group (*p* < 0.001); Compared with the 5/6 nephrectomy group, HDHW and AS-IV significantly downregulated the expression of Beclin1 and LC3II/Ⅰ (*p* < 0.001), and there was no difference between HDHW and AS-IV groups (*p* > 0.05) (Figures 4A,C,D). Western blot analysis also showed that HDHW and AS-IV could significantly down-regulate the expressions of Beclin1 and LC3II/Ⅰ (*p* < 0.05) (Figures 4B,E,F).

### HeidihuangWan affects the expression of IGF-1-mediated PI3K/Akt/mTOR signaling pathway

**FIGURE 5:**
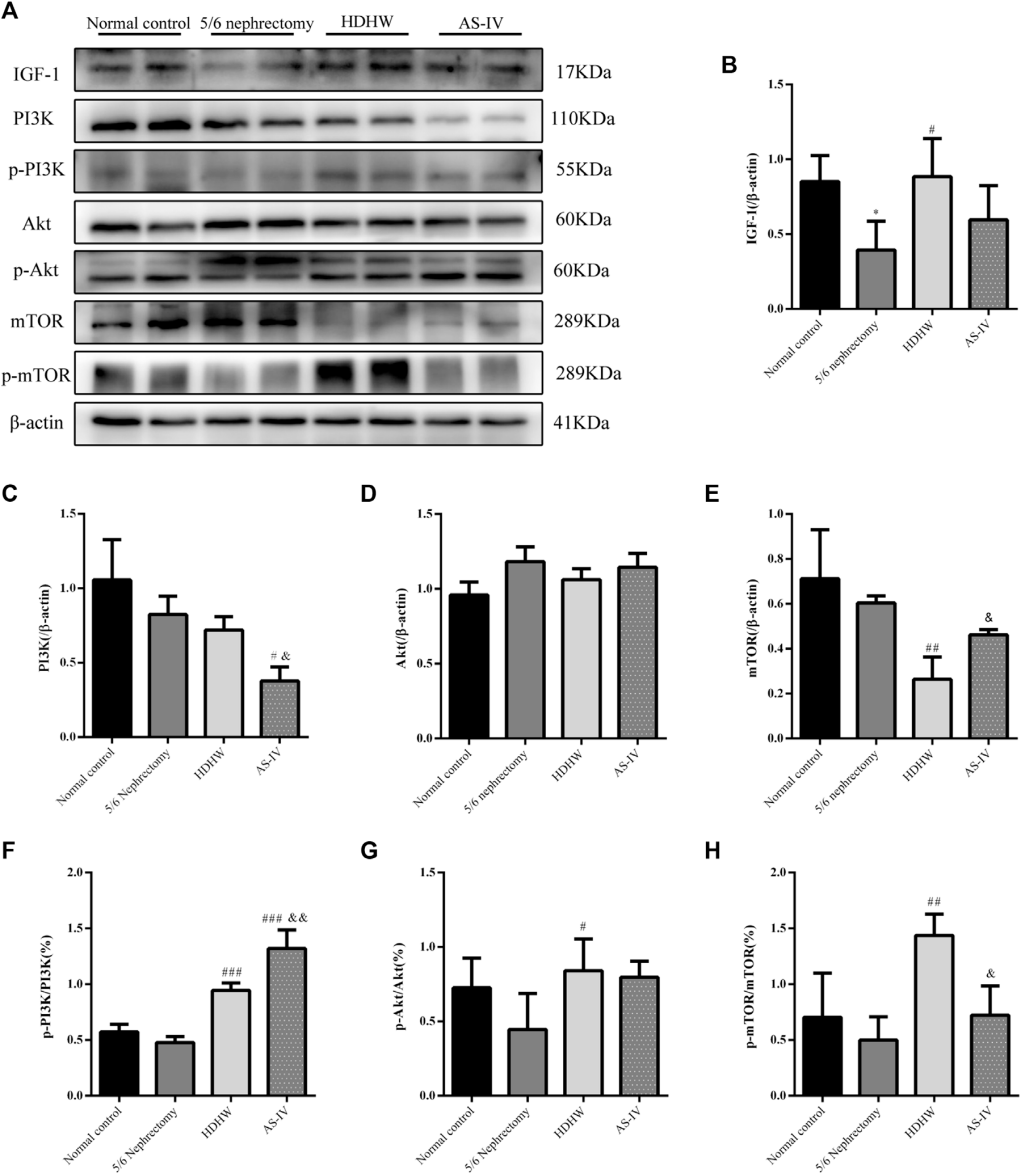
Effects of HeidihuangWan (HDHW) on IGF-1-mediated PI3K/Akt/mTOR pathway. **(A)** Western Blotting to detect the protein levels of IGF-1, PI3K, p-PI3K, Akt, p-Akt, mTOR, and p-mTOR. **(B,C,D,E,F,G,H)** Protein concentration analysis. Data are presented as mean ± SD, *n* = 3. The data are expressed as *p < 0.05: compared with normal control group; #*p* < 0.05, ##*p* < 0.01, ##*p* < 0.001: compared with 5/6 nephrectomy group; &*p* < 0.05, &&*p* < 0.01: compared with HDHW group, respectively.

The PI3K/Akt/mTOR pathway is a key pathway that regulates autophagy, and activation of the PI3K/Akt/mTOR pathway can inhibit autophagy. IGF-1 is an upstream regulatory molecule of the PI3K/Akt/mTOR pathway. We examined the expression of IGF-1, PI3K, p-PI3K, Akt, p-Akt, mTOR, and p-mTOR in rat kidneys. The results showed that the expression of IGF-1 was down-regulated in the 5/6 nephrectomy group, compared with the normal control group (*p* < 0.05); HDHW up-regulated the expression of IGF-1, compared with the 5/6nephrectomy group (*p* < 0.05) (Figures 5A,B). Compared with the 5/6 nephrectomy group, the expression of PI3K and Akt in the HDHW group had no significant change (*p* > 0.05). However, the expression of mTOR was significantly down-regulated (*p* < 0.01), while the expressions of p-PI3K/PI3K, p-Akt/Akt, and p-mTOR/mTOR were significantly up-regulated (*p* < 0.05) (Figures 5A,C–H), indicating that HDHW activated IGF-1/PI3K/Akt/mTOR pathway. This may be the mechanism by which HDHW regulates autophagy and anti-nephrogenic fibrosis.

### HeidihuangWan inhibits autophagy by regulating IGF-1-mediated PI3K/Akt/mTOR pathway and exerts anti-fibrosis effect

**FIGURE 6:**
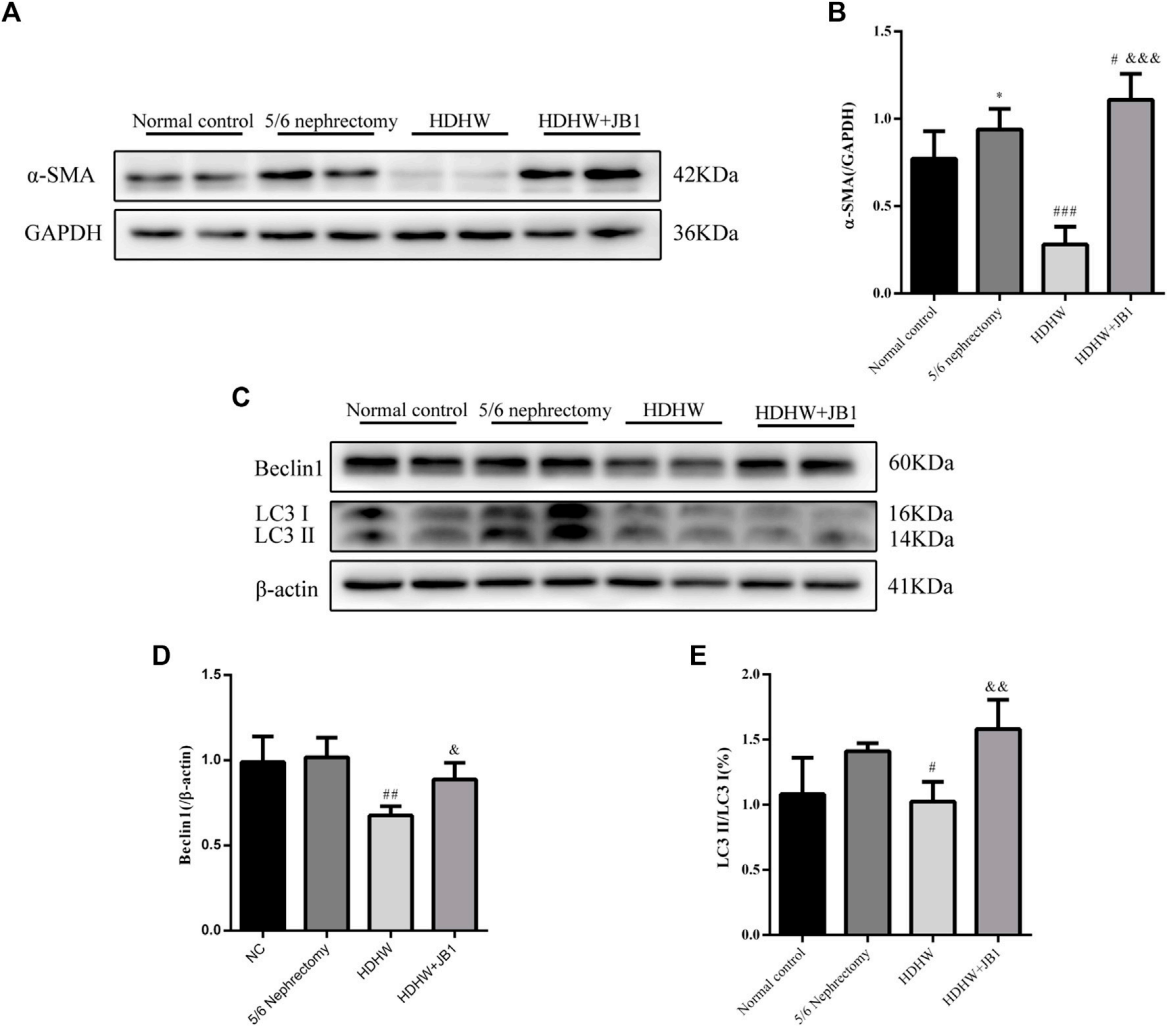
IGF-1R inhibitor (JB1) blocks the inhibitory effect of HeidihuangWan (HDHW) on renal fibrosis and autophagy. **(A,C)** Western Blotting for protein levels of α-SMA, Beclin1, LC3II. **(B,D,E)** Protein concentration analysis. Data are presented as mean ± SD, *n* = 3. The data are expressed as *p < 0.05: compared with normal control group; #*p* < 0.05, ##*p* < 0.01, ###*p* < 0.001: compared with 5/6 nephrectomy group; &*p* < 0.05, &&*p* < 0.01, &&&*p* < 0.001: compared with HDHW group, respectively.

**FIGURE 7:**
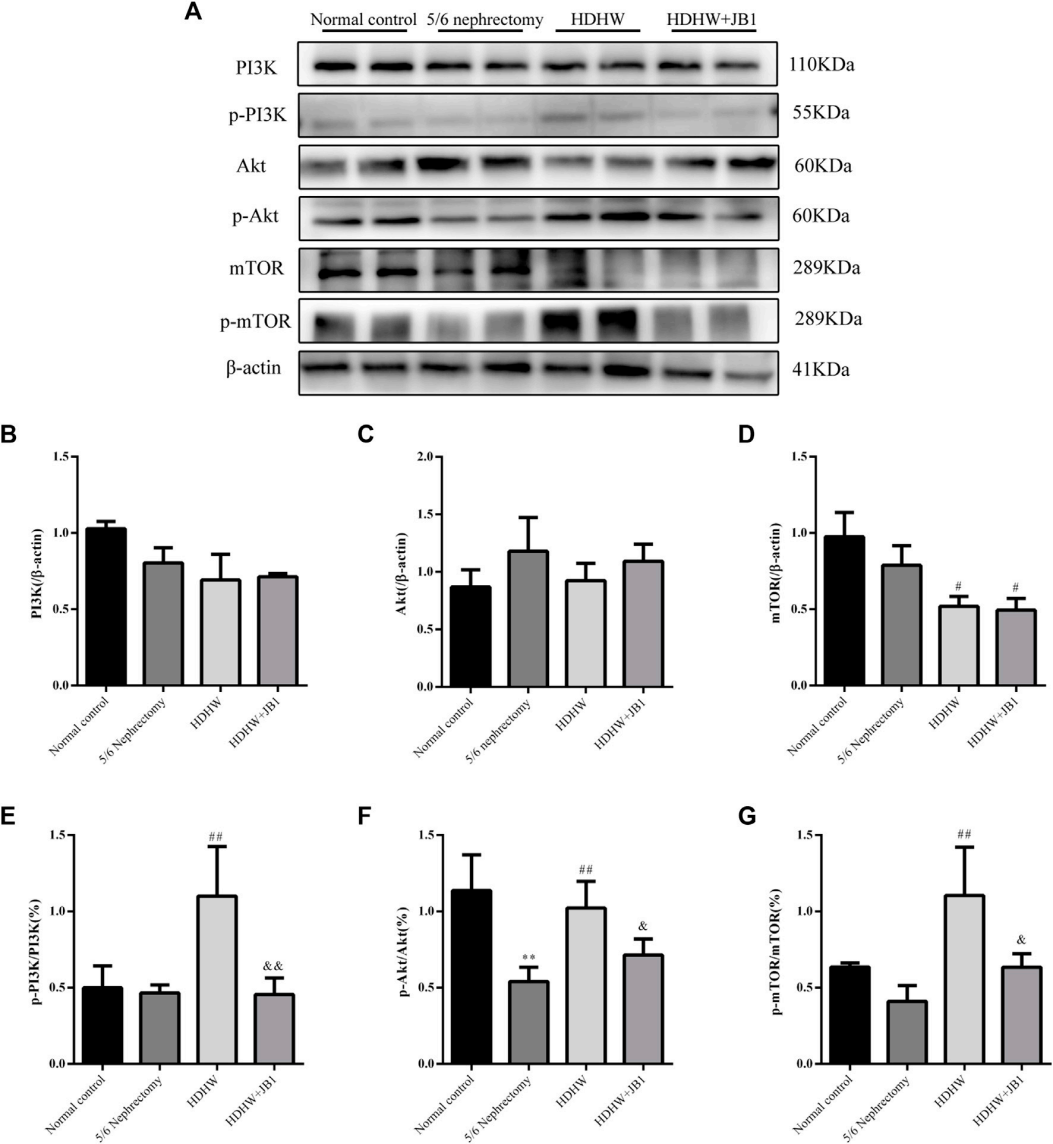
HeidihuangWan (HDHW) exerts nephroprotective effects by regulating the IGF-1-mediated PI3K/Akt/mTOR pathway. **(A)** Western Blotting for protein levels of PI3K, p-PI3K, Akt, p-Akt, mTOR, p-mTOR. **(B,C,D,E,F,G)** Protein concentration analysis. Data are presented as mean ± SD, *n* = 3. The data are expressed as **p < 0.01: compared with normal control group; #*p* < 0.05, ##*p* < 0.01: compared with 5/6 nephrectomy group; &*p* < 0.05, &&*p* < 0.01: compared with HDHW group, respectively.

In order to further verify that the regulation of autophagy and the anti-fibrosis effect of HDHW is related to the IGF-1-mediated PI3K/Akt/mTOR pathway, we blocked the binding of IGF-1 to the IGF-1R receptor. Masson staining analyzed the amount of collagen fiber deposited; Western blot was used to detect the expression of fibrosis marker protein α-SMA, autophagy marker protein Beclin1, LC3Ⅱ, and the expression of PI3K/Akt/mTOR signaling pathway proteins. The results showed that the deposition of collagen fibers in HDHW+JB1 group was significantly more than that in HDHW group (*p* < 0.01) (Figure 2E). Meanwhile, the expression of α-SMA was significantly up-regulated in the HDHW+JB1 group compared with the HDHW group (*p* < 0.001) (Figures 6A,B), indicating that the effect of HDHW in reducing renal fibrosis was related to the regulation of IGF-1 signaling. Compared with the HDHW group, the expressions of Beclin1 and LC3II/LC3I in the kidney tissue of the HDHW+JB1 group were significantly up-regulated (*p* < 0.05) (Figures 6C–E), indicating that the inhibitory effect of HDHW on autophagy was related to the regulation of IGF-1 signaling. Compared with HDHW group, PI3K, Akt and mTOR in kidney tissue of HDHW+JB1 group had no significant changes (*p* > 0.05) (Figures 7A–D), but the expression of p-PI3K/PI3K, p-Akt/Akt, and p-mTOR/mTOR were significantly down-regulated (*p* < 0.05) (Figures 7A,E–G), indicating that HDHW activates the PI3K/Akt/mTOR pathway by promoting the binding of IGF-1 to IGF-1R.

## Discussion

It has been shown that single herbs (Liu et al., 2022; Qian et al., 2022), herbal extracts (Yu, 2020; Zheng et al., 2021; Cong et al., 2022; Ma et al., 2022), and Chinese medicine compounds (Chen et al., 2022a; Chen et al., 2022b; Wu et al., 2022; Zhao et al., 2022) have made great progress in the prevention and even treatment of renal fibrosis (Xu et al., 2021). They regulate multiple signaling pathways such as Wnt/β-catenin (Zhao et al., 2022), TGF-β1/Smad (Mao et al., 2019; Wu et al., 2022), Akt/mTOR (Lian et al., 2022), CX3CL1-RAF/MEK/ERK (Hu et al., 2022b), mTORC1/p70S6K (Chen et al., 2019), SIRT1-NF-κB (Wang et al., 2018b), and interfere with different cytokine or inflammatory factors expression to regulate the development and progression of renal fibrosis. The Chinese herbal extract AS-IV, which has been widely recognized to have significant anti-nephrogenic effects (Yu, 2020; Fu et al., 2022), is the main active component of Huangqi, a Chinese herbal medicine, and its mechanism has been richly studied and repeatedly validated (Wang et al., 2018b; Chen et al., 2019; Mao et al., 2019; Hu et al., 2022b; Lian et al., 2022). Therefore, it was used as a positive control drug in this study. Traditional Chinese medicine (TCM) is a rich source of drug discovery with its multi-component, multi-target, and multi-pathway characteristics, which can enhance body functions and reduce drug toxicity through the synergistic effects of its main active ingredients. Studying the specific mechanism of TCM in the treatment of renal fibrosis can identify more potential targets for the treatment of renal fibrosis (Zhang et al., 2022b).

HDHW is a traditional Chinese medicine compound formula containing 4 herbs, including Shu Di Huang, Cang Zhu, Gan Jiang, and Da Zao. My supervisor has been working on the efficacy and mechanism of HDHW in the treatment of kidney diseases for more than 10 years. The good clinical efficacy and significant renal protection effect of HDHW prompted us to further study the mechanism of its renal protection. In this study, HDHW components were identified by UHPLC-Q-Orbitrap-MS/MS and 14 components were identified. Among these 14 ingredients, the anti-renal fibrosis effects of Linoleic acid (Zhan et al., 2015; Zhou et al., 2017), Oleanolic acid (Zhao and Luan, 2020), Nicotinamide (Kumakura et al., 2021), Rutin (Han et al., 2015; Wang et al., 2016), Gallic acid (Yousuf and Vellaichamy, 2015), Ferulic acid (Mir et al., 2018; Qi et al., 2020), Fraxetin (natural derivatives of coumarin) (Hsieh et al., 2021), and Theophylline (adenosine antagonist) (Tang et al., 2015) have been demonstrated. They exert antifibrotic effects mainly through autophagy regulation, apoptosis regulation, antioxidant, and anti-inflammatory. The identification of HDHW components provides additional evidence to support its anti-nephrogenic effects. In addition, we also detected the levels of Scr and BUN in rats and observed the renal pathological changes by HE, PAS, and Masson staining, and confirmed that HDHW significantly improved renal function in 5/6 nephrectomized rats, reduced atrophy of proximal tubules, infiltration of inflammatory cells, collagen deposition, and reduced fibrous tissue proliferation, which verified the renal protective effect of HDHW. Moreover, we also confirmed the inhibitory effect of HDHW on renal fibrosis from three indicators, α-SMA, Vimentin, and TGF-β, which we have never observed before. Interestingly, we found that HDHW also significantly reduced the expression of TGF-β in the kidneys of 5/6 nephrectomized rats and was more potent than the positive control drug AS-IV. TGF-β is an important molecule that accelerates renal fibrosis (Chen et al., 2022b). This suggests that TGF-β may also be a pathway for HDHW to improve renal fibrosis, but we did not continue to study the effect of HDHW on TGF-β-mediated pathways in this article.

Autophagy is closely associated with renal fibrosis (Tang et al., 2020). In the kidney, basal autophagy of renal cells is critical for maintaining renal homeostasis, structure, and function. In contrast, under stressful conditions, autophagy is altered as part of an adaptive response in kidney cells, a process that is tightly regulated by key regulators of autophagy, the mammalian target of rapamycin, AMP-activated protein kinases and deacetylases, and related signaling pathways. Activation of autophagy protects kidney cells under stress, while lack of autophagy increases kidney susceptibility to injury, leading to impaired renal function and accumulation of damaged mitochondria, which in turn leads to more severe renal fibrosis. However, other studies have found that persistent activation of autophagy is detrimental after severe renal injury, leading to renal cell senescence and promoting renal fibrosis by secreting pro-fibrotic cytokines (Liang et al., 2022). Therefore, maintaining the balance of autophagy is crucial for improving renal fibrosis.

Several researchers have pointed out that in certain diseases, the role of autophagy depends on the type of cells involved in the disease process and the stage of the disease (Tang et al., 2020). For example, under physiological conditions, podocytes have higher levels of basal autophagy (Bork et al., 2020) and renal tubular epithelial cells have lower levels of autophagy (Liu et al., 2012). In an ischemia-reperfusion-induced acute kidney injury mouse model, autophagy is enhanced in the proximal tubule after 1 day of reperfusion and is eliminated by fusion of autophagosomes with lysosomes on day 3 (Li et al., 2014; Cheng et al., 2015). These findings suggest that autophagy increases cell survival during the initial tubular injury but impedes normal renal repair during the recovery period. Some findings suggest a protective role for autophagy in renal fibrosis. For example, in a study of the anti-RIF mechanism of curcumin, the number of autophagosomes was reduced and LC3II and Beclin1 protein expression was decreased in the UUO rat model of renal interstitial fibrosis. At the same time, PI3K/Akt signaling pathway was activated and mTOR was upregulated. In contrast, curcumin reversed this process and attenuated RIF (Lu et al., 2021). The UUO mouse model exhibited mitochondrial damage, ROS production, TGF- β1/Smad pathway activation, epithelial-mesenchymal transition, and renal fibrosis, and these changes were ameliorated by the use of UMI-77 (mitochondrial autophagy activator) (Jin et al., 2022), suggesting that activation of autophagy protects the damaged kidney. In adult Sprague Dawley rats, the expression of autophagy marker proteins Beclin1, LC3II, PRR, ATG7, and ATG5 was significantly lower in glomeruli than in normal rats 8 weeks after undergoing 5/6 nephrectomy (Yildirim et al., 2021). In addition, a high phosphorus diet increased renal impairment and interstitial fibrosis in 5/6 nephrectomized Wistar rats, a process that was shown to be associated with inhibition of autophagy (Duayer et al., 2021). On the other hand, there is also evidence to support that autophagy activation promotes renal fibrosis. d. Zepeda-Orozco et al. (Li et al., 2010) first identified enhanced autophagy in the obstructed kidney of the UUO mouse model, including accumulation of autophagosomes, increased expression of Beclin 1, and increased conversion of LC3I to II. Another study using UUO mice as a model of renal fibrosis came to similar conclusions, with increased Beclin 1 and LC3 expression and decreased P62 expression. At the same time, the levels of fibronectin, type I collagen, and α-SMA were significantly increased. However, injection of the autophagy inhibitor ODN significantly decreased the expression of Beclin1 and LC3 and reduced the expression of renal fibrosis marker proteins. This suggests that by inhibiting autophagy, renal fibrosis can be attenuated (Wu et al., 2021; Jung et al., 2022). In the UUO rat model, rats were executed on days 3, 7, and 14 after modeling, and time-dependent induction of autophagy was found in both the obstructed and contralateral unobstructed kidneys, and sustained activation of autophagy led to tubular apoptosis and renal fibrosis, whereas the autophagy inhibitor 3-methyladenine (3-MA) inhibited sustained autophagy-induced tubular apoptosis and renal fibrosis (Kim et al., 2012). Further *in vitro*, experimental studies confirmed that inhibition of mTOR in some proximal tubular cells resulted in sustained activation of autophagy and impaired proliferation of proximal tubular cells. Activation of mTOR inhibits autophagy and contributes to renal tubular repair (Li et al., 2014).

An interesting finding is that the role of autophagy in renal fibrosis differs in different models of renal fibrosis (Li et al., 2010; Lu et al., 2021; Yildirim et al., 2021); In the same model of renal fibrosis (Lu et al., 2021) (Kim et al., 2012), the role of autophagy in renal fibrosis differs even when the time points of intervention are essentially the same. The former may be related to the different pathological changes of renal injury induced by different modeling methods, such as the pathological changes in the 5/6 nephrectomy model: increased glomerular extramural matrix, a proliferation of thylakoid cells, capillary dilatation or occlusion, focal or total glomerular sclerosis, tubular atrophy or dilatation, massive protein tubular pattern, increased renal interstitium, and diffuse infiltration of inflammatory cells; The pathological changes in the UUO model are: renal interstitial collagen fiber hyperplasia, diffuse infiltration of inflammatory cells, tubular atrophy or dilation; glomerular basement membrane thickening, glomerular glassy changes (Ma et al., 2018). The latter requires more studies to reveal and justify. In conclusion, the relationship between autophagy and renal fibrosis needs more studies to reveal and prove. Unfortunately, our immunohistochemical results showed that the level of autophagy was elevated in the kidneys of 5/6 nephrectomized rats, supporting a pathological role of autophagy in renal injury. However, our Western Blot results showed that the autophagy level of 5/6 nephrectomy rats showed an upward trend compared with normal rats of the same age, but it was not statistically significant. Therefore, increasing the sample size of the study is necessary to further confirm the level of autophagy in the kidneys of 5/6 nephrectomized rats.

It has been shown that herbal medicines can exert antifibrotic effects by modulating autophagy (Ma et al., 2018). Cytoprotective effects of autophagy regulators have so far only been reported in animal models of kidney disease, and evidence that these findings can be applied to humans is currently lacking (Tang et al., 2020). The results of this study showed that HDHW could significantly down-regulate the expressions of Beclin1 and LC3II/I in the kidneys of 5/6 nephrectomized rats, suggesting that the anti-renal fibrosis of HDHW may be related to the inhibition of autophagy. Therefore, HDHW may be a promising regulator of autophagy. Further study revealed that HDHW can significantly up-regulate the expression of IGF-1 in the kidney of 5/6 nephrectomy rats, and at the same time significantly up-regulate the expressions of p-PI3K, p-Akt, and p-mTOR. This suggests that HDHW activates the IGF-1/PI3K/Akt/mTOR signaling pathway, a key pathway in regulating autophagy (Tang et al., 2020), which further supports the regulatory effect of HDHW on autophagy in renal fibrosis rats. To verify that the regulation of autophagy and the improvement of renal fibrosis by HDHW are related to the IGF-1-mediated PI3K/Akt/mTOR signaling pathway, this study blocked the binding of IGF-1 to IGF-1R by intraperitoneal injection of IGF-1R blocker JB1 and observed the expression of downstream signaling factors, including PI3K, Akt, mTOR, p-PI3K, p Akt, p-mTOR, as well as the expression of autophagy marker proteins including Beclin1, LC3II/Ⅰ, and renal fibrosis marker proteins including α-SMA. The results of the study showed that the inhibitory effect of HDHW on autophagy and renal fibrosis was altered after blocking the binding of IGF-1 to IGF-1R, and the phosphorylation processes of PI3K, Akt, and mTOR were also blocked. This result confirms that HDHW can inhibit autophagy and attenuate renal fibrosis by upregulating IGF-1 expression, promoting the binding of IGF-1 to IGF-1R, and activating the autophagy-related pathway PI3K/Akt/mTOR. In addition, HDHW has been widely used in the clinical treatment of chronic kidney disease, especially chronic renal failure, with good therapeutic effects, and the autophagy-regulating effect of HDWH on renal fibrosis was found in this study, providing a reference for the lack of autophagy regulators for human use (Tang et al., 2020).

## Conclusion

**FIGURE 8:**
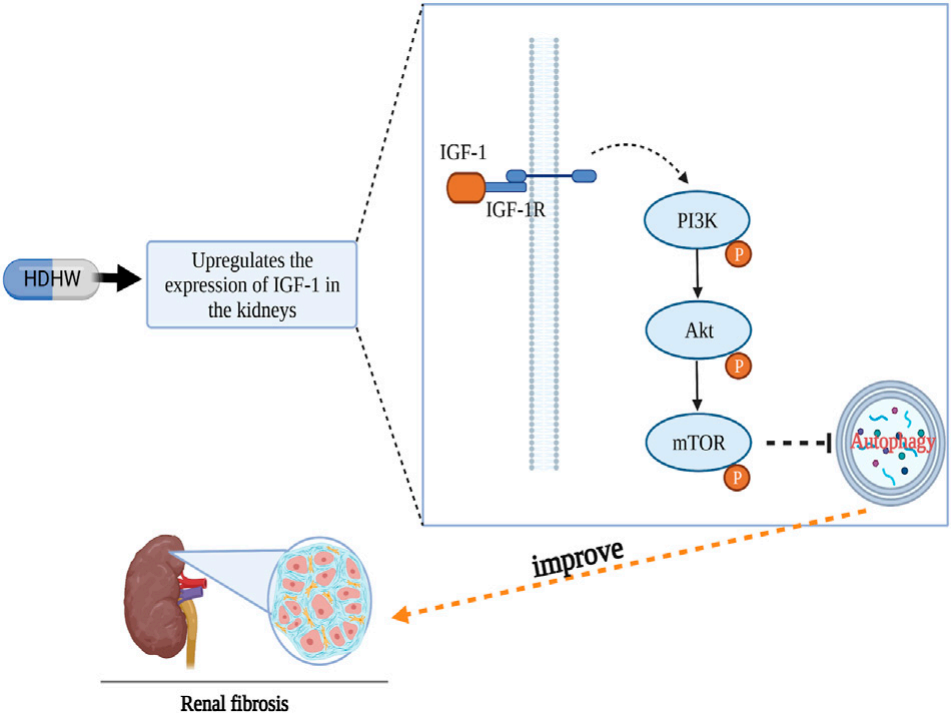
Diagram of the anti-renal fibrosis mechanism of HeidihuangWan.

In conclusion, our results showed that HDHW improved renal function and renal pathological damage and attenuated renal fibrosis in 5/6 nephrectomized rats. The mechanism of HDHW ameliorating renal fibrosis may be that HDHW inhibits autophagy by upregulating IGF-1 expression, promoting the binding of IGF-1 to IGF-1R, and activating the autophagy-related pathway PI3K/Akt/mTOR, which in turn attenuate renal fibrosis (Figure 8).

## Data Availability

The original contributions presented in the study are included in the article/supplementary material, further inquiries can be directed to the corresponding authors.

## Abbreviations

CKD, Chronic kidney diseases; HDHW, HeidihuangWan; AS-IV, Astragaloside IV; Scr, Serum creatinine; BUN, blood urea nitrogen; HE, Hematoxylin-Eosin; PAS, Periodic Acid-Schiff; Masson, Modified Masson’s Trichrome; α-SMA, α-smooth muscle actin; TGF-β, Transforming growth factors-β; PI3K, class I phosphatidylinositol-3 kinase; Akt, serine-threonine kinase; IGF-1, Insulin-like growth factor 1; IGF-1R, Insulin-like growth factor 1 receptor; ESRD, End-stage renal disease; CRF, Chronic renal failure; EMT, epithelial-mesenchymal transition; ECM, extracellular matrix; NF-κB, nuclear factor-kappaB; Wnt, wingless-type MMTV integration site; PKC, protein kinase C; ERK, extracellular regulated protein kinases; TCMs, Traditional Chinese medicines; RIF, Renal interstitial fibrosis; UHPLC-Q-Orbitrap-MS/MS, Ultra High Performance Liquid Chromatography-Quadrupole-Electrostatic Field Orbitrap Tandem Mass Spectrometry.
